# Multisource feedback as part of the Medical Board of Australia’s Professional Performance Framework: outcomes from a preliminary study

**DOI:** 10.1186/s12909-018-1432-7

**Published:** 2018-12-29

**Authors:** Ajit Narayanan, Elizabeth A. Farmer, Michael J. Greco

**Affiliations:** 1 Computer and Mathematical Sciences, School of Engineering, Auckland University of Technology, 2-14 Wakefield Street, Auckland, 1010 New Zealand; 2 Graduate Medicine, University of Wollongong NSW, Keiraville, Australia; 3 School of Medicine, Gold Coast Campus, Griffith University, Southport, Australia

**Keywords:** Multisource feedback, Self-evaluation, Professional performance, Continuing professional development

## Abstract

**Background:**

The recent introduction of the Professional Performance Framework by the Medical Board of Australia is intended to strengthen continuing professional development for the 100,000 or so medical practitioners in Australia. An important option within the Framework is the use of multisource feedback from patients, colleagues and self-evaluations to allow doctors to reflect on their performance and identify methods for self-improvement. The aim of this study is to explore the relationships between patient feedback, colleague feedback, and self-evaluation using the same questionnaires as used by patients and colleagues.

**Methods:**

Feedback data for around 2000 doctors belonging to four different groups were collected through non-probability sampling from nearly 100,000 patients and 24,000 colleagues. Reliability analysis was performed using single measures intraclass coefficients, Cronbach’ alpha and signal-to-noise ratios. Analysis of variance was used to identify significant differences in scores between items and sub-populations of doctors; principal component analysis involving Kaiser-Meyer-Olkin (KMO) sampling adequacy and Bartlett’s test for sphericity was used to reveal components of doctor performance; and correlation analysis was used for identifying convergence between sets of scores from different sources.

**Results:**

Patients rated doctors highest on respect shown and lowest on reassurance provided. Colleagues rated doctors highest on trustworthiness and lowest on ability to say ‘no’. With regard to self-evaluation, doctors gave themselves lower scores on the patient questionnaire and the colleague questionnaire (10 and 12%, respectively) than they received from their patients and colleagues. There were weak but positive correlations between self-scores and scores received indicating some convergence of agreement, with doctors feeling more comfortable with self-evaluation from the perspective of patients than from colleagues.

**Conclusions:**

Supplementing patient and colleague feedback with self-evaluation may help doctors confirm for themselves areas for enhanced CPD through convergence. If self-evaluation is used, the colleague questionnaire may be sufficient, since aspects of clinical competence, management, communication and leadership as well as patient care can be addressed through colleague items. Mentoring of doctors in CPD should aim to make doctors feel more comfortable about being rated by colleagues to enhance convergence between self-scores and evaluations from the perspective of colleagues.

## Background

Medical regulatory authorities continue to develop guidelines and frameworks for ensuring that doctors perform competently in response to growing legal and statutory requirements regarding licensure and accreditation in many countries [1, 2]. The need for assessment methods that help doctors to demonstrate continued professional competence which are as rigorous as those used for initial licensure is now accepted as a desirable objective. However, questions remain about whether these methods should be voluntary or regulatory [3]. Frameworks for establishing doctor competencies in medical schools internationally are showing signs of consolidation [4], with doctors themselves identifying the need for newly-trained colleagues to demonstrate professional behaviour (e.g. dependability, integrity, stress tolerance, cooperation) and professional values (e.g. achievement, leadership, initiative, social orientation) in addition to clinical competence and knowledge of technical procedures [5]. In some countries, doctors are legally obliged to maintain their professional competence through various professional development schemes in accordance to national medical council requirements [6]. In the UK, for example, the GMC has implemented its own 5-year revalidation scheme for all its licensed doctors using a set of ‘good medical practice’ guidelines [7].

Recently, the Medical Board of Australia (MBA), after considering issues of revalidation [8], has issued its latest version of the Professional Performance Framework (PPF) for strengthening continuing professional development (CPD) for its 100,000 or so registered medical practitioners [9]. One of the core features of the MBA’s PPF is the allocation of minimum CPD requirements across three types of activity: educational activity to develop knowledge and skills; activities focused on reviewing performance; and activities on measuring outcomes. Activities focused on reviewing performance may include peer review of performance, performance appraisal, peer review of medical records, peer discussion of cases, peer review of journal articles and peer review of educational activities. One other key performance review activity, which is the focus of this paper, is multisource feedback (MSF) from peers, medical colleagues, co-workers, patients and other health practitioners [8].

MSF is playing an increasingly important role in continuing revalidation, re-licensure and CPD activities, with several schemes requiring their use to help doctors reflect on how they work and identify ways for self-improvement [10]. Feedback from colleagues and patients has been well-established in personal development planning processes in many countries for several years, including the UK and Australia [11, 12].

Other areas of application include use in training, especially when merged with self-evaluation to MSF [13–15]. Such MSF was initially based on patient and colleague feedback but more recently MSF has started to incorporate doctor self-evaluation for the purposes of promoting reflection on personal performance and identifying reasons for discrepancies between received scores and self-scores [16–18]. Self-evaluation can employ the same questionnaires as used by patients and colleagues, with doctors rating themselves from these alternative perspectives. The dominant approach for obtaining patient and colleague feedback is now through questionnaires in which raters are asked to give their evaluations of a doctor on multi-point Likert-scale items [19, 20]. MSF can therefore be interpreted in three ways: 180° MSF (patient and colleague feedback through specially designed patient and colleague questionnaires); 270° feedback (patient and colleague feedback with self-evaluation through one of the questionnaires); and 360° feedback (patient and colleague feedback with self-evaluation through both questionnaires).

Over time, the amount and range of MSF studies have increased to the point where some general trends are starting to emerge [21, 22], including the interesting possibility that self-evaluation may not be correlated with patient or colleague ratings [23]. The measurement relationship between scores given by patients or colleagues and self-evaluation scores is not known with any certainty. Any mismatches between external review scores and self-review scores, if they are to be acted on for CPD, must not be due to the unreliability of the instruments or methods used for collecting the data.

The aim of this study is to test the reliability and validity of two MSF questionnaires and the data derived from them, and to identify how doctors can use such data for professional development purposes, as exemplified in the MBA’s recently published PPF. In particular, there is a need to understand the dimensions along which mismatches can occur so that future feedback and self-evaluation mechanisms can address and correct for those mismatches as part of the CPD process.

The patient questionnaire deals with the patient’s visit to their doctor and asks patients to rate their experience using 10 performance-based questions (questions asking patients to rate specific ways that their doctor behaved towards them) and 2 summative questions (questions asking for an overall impression of their visit). The colleague questionnaire asks colleagues to rate their interactions with the doctor using 20 questions, with 19 of these questions dealing with clinical competence, management, communication and leadership. There is a final summative question relating to overall ability. All items use a five point Likert scale with labels ‘poor’, ‘fair’, ‘good’, ‘very good’, and ‘excellent’. The patient and colleague items are described in concise form in Tables 1 and 3, respectively.

**Table 1:** Overview of all 14 items on the patient questionnaire (total *n* = 98,305)

Patient items	*N*	Mean	Std. Deviation	Missing	
				Count	Percent
Q1 Satisfaction with visit	98,107	88.24	14.961	198	.2
Q2 Warmth of greeting	98,064	89.94	14.143	241	.2
Q3 Ability to listen	97,960	89.87	14.271	345	.4
Q4 Explanations	97,854	88.70	14.850	451	.5
Q5 Reassurance	97,829	87.79	15.396	476	.5
Q6 Confidence in ability	97,843	88.04	15.184	462	.5
Q7 Express concerns	97,673	88.61	14.901	632	.6
Q8 Respect shown	97,931	91.61	13.227	374	.4
Q9 Time for visit	97,920	88.80	14.961	385	.4
Q10 Consideration	97,663	89.21	14.733	642	.7
Q11 Concern for patient	97,932	89.62	14.382	373	.4
Q12 Recommendation	97,773	89.70	14.904	532	.5
*Averages*	*97,879.08*	*89.16*	*14.66*	*425.92*	*0.43*

The research strategy adopted here is to first establish the validity and reliability of the patient and colleague questionnaires and data through exploratory principal component analysis (PCA) and other measures. The second task is to identify convergent and divergent relationships between doctors’ evaluations of themselves using the patient and colleague questionnaires and how these evaluations differ from those provided by their patients and colleagues. Analysis of variance (ANOVA), correlations and PCA are used in the second phase of analysis. Further statistical details can be found in the Statistical Analysis section below.

## Methods

### Data collection.

Data collection was during the period 2013–2016 and varied across the four doctor groups during this period.

- Group 1 consists of registrars belonging to the Royal Australian College of General Practitioners (‘RACGP’ below) whose data were gathered from those Regional Training Organisations (RTOs) who require their registrars to undertake an MSF as part of their vocational training requirements. Not all RTOs require their registrars to undertake MSF.
- Group 2 consists of GPs undertaking MSF as a CPD activity (‘GPs-CPD’). This is voluntary and doctors in this group directly approached CFEP Surveys to undertake MSF.
- Group 3 consists of registrars mandated by Australian College of Rural & Remote Medicine (‘ACRRM’) to undertake MSF.
- Group 4 consists of a subgroup of doctors required by Australian Health Practitioner Regulation Agency (‘AHPRA’) to undertake MSF as part of a regulatory requirements to help them progress to provisional registration.

The patient questionnaire is a post-consultation exit survey. Practice staff were advised to hand out questionnaires to consecutive patients for each participating doctor. Patients were requested to complete the questionnaire following their consultation and to rate their experience according to their satisfaction with that specific visit. To ensure patient confidentiality and to encourage honest feedback, envelopes for completed questionnaires were provided. Patient anonymity was guaranteed at all stages of the survey process. The colleague questionnaire, on the other hand, was completed online or as a paper postal survey. To provide the most representative overall picture of performance, participating doctors were advised to nominate a range of colleagues with whom they work, to include doctors, other healthcare professionals and managerial/administrative staff. Nominated colleagues were then sent the questionnaire for completion, with a follow up reminder if required. Colleague anonymity was guaranteed for all responses provided. Brief descriptions of the questionnaire items are presented in Tables 1 and 3. Further details concerning the content and format of the two questionnaires can be obtained by emailing the authors.

Questionnaires were processed by CFEP Surveys (a professional health survey organisation) in Brisbane, Australia. Paper questionnaires were scanned and verified electronically by an experienced data auditor. Data were imported to an in-house software system running on an enterprise database where they were further checked and verified. The colleague online questionnaires were completed via a secure online web portal. Online validation and verification were conducted before being downloaded to in-house software systems; the same procedures were then carried out as for the paper questionnaires. The patient and colleague datasets were exported as Microsoft Excel Spreadsheets to an SPSS database (SPSS for Windows Version 23.0) and cleaned and checked prior to data analysis.

Doctor self-evaluations using the colleague questionnaire were collected from about half-way through the data collection process and using the patient questionnaire only towards the end, leading to varying numbers of doctors with self-evaluations. In total, four sets of data were obtained as follows for the analysis reported here:

- from a patient questionnaire consisting of 98,305 patient responses to 2449 doctors (1564 RACGP Registrars; 95 GPs-CPD; 506 ACRRM Registrars; 284 AHPRA doctors);
- from a colleague questionnaire consisting of 23,268 colleague responses to 1890 doctors (1044 RACGP Registrars; 15 GPs-CPD; 546 ACRRM Registrars; 285 AHPRA doctors);
- from a self-evaluation by 375 doctors using the patient questionnaire (109 RACGP Registrars, 266 AHPRA doctors); and
- from a self-valuation by 1888 doctors using the colleague questionnaire (1042 RACGP Registrars, 14 GPs-CPD, 547 ACRRM Registrars, 285 AHPRA doctors).

**Fig. 1:**
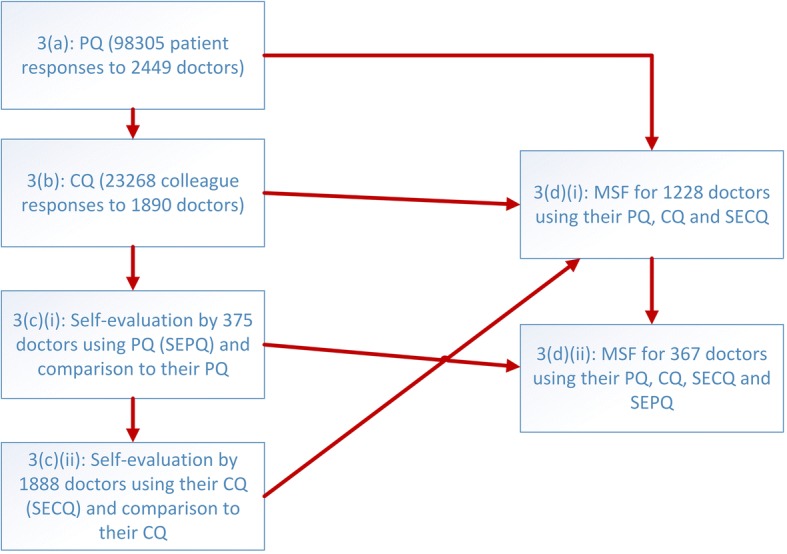
Overview of data analysis methodology in Sections 3(a) to 3(d). Patient questionnaires (PQ), colleague questionnaires (CQ) and self-evaluation using patient (SEPQ) and colleague (SECQ) questionnaires are first analysed separately (left part of methodology) before being combined into two forms of multi-source feedback (MSF) for doctors (right part)

Each of these data sets was analysed separately, then combined first through patient and colleague data and finally by self-evaluation using the patient questionnaire and colleague questionnaire. Figure 1 provides an overview of the data analysis methodology adopted for the Results section 3 below.

### Statistical analysis

On the basis that the intervals between the five Likert scale points used in the questionnaires are equal, all responses were converted into percentages (‘poor’ = 20%, ‘fair’ = 40%, ‘good’ = 60%, ‘very good’ = 80%, ‘excellent’ = 100%) to allow for parametric techniques based on means, standard deviations and variances. Two levels of analysis were conducted: at the raw score rater and item level (irrespective of doctor rated), and at the aggregated doctor level where doctors received the average item scores of all their raters.

Non-probability (convenience) sampling, as used in this study for collecting patient and colleague data, involves participants who are available and willing to take part in the research. Such sampling also implies that the data collection takes place at the location where it makes sense to seek participants’ views, in this case, the primary care health centre and immediately after a consultation for patients, and in the doctor’s clinical environment for colleagues. The data from non-probability sampling will have special characteristics (unbalanced because of variable numbers of raters per ratee, fully nested because all the ratees may be unique to that rater, and uncrossed because ratees provide only one rating per ratee on one occasion).

Analysis of variance (ANOVA) is a collection of statistical models used to analyze the differences among group means. The observed variance in a particular variable is partitioned into components attributable to different sources of variation. In its simplest form, ANOVA provides a statistical test of whether or not the means of several groups are equal.

Principal component analysis (PCA) is a data reduction technique for explaining variance in data using a smaller set of variables than the original variables or items. It is a statistical procedure that uses an orthogonal transformation to convert a set of observations of possibly correlated variables into a set of values of linearly uncorrelated variables called principal components. Exploratory PCA uses PCA to discover component structures formed from different items. Varimax method is used in this report for rotating and extracting the components, whereby each component has a small number of large loadings. The Kaiser-Meyer-Olkin (KMO) test is a sampling measure to determine common variance among variables, with lower proportions indicating higher suitability for factor analysis and PCA. KMO values between 0.8 and 1.0 indicate that there are enough samples and sufficiently low variance for efficient identification of any components through exploratory PCA. Bartlett’s test for sphericity is another measure for testing the suitability of data reduction which check for correlations between variables. A significant Bartlett test indicates the variables are sufficiently correlated for PCA.

Single measures intraclass coefficients (ICCs) provide a relative measure of the variability in the sample of responses and is useful for estimating the agreement between raters on how to interpret the items. Values between 0.4 to 0.6 are considered ‘moderate agreement’, between 0.6 and 0.8 ‘good agreement’ and above 0.8 ‘very good agreement’ [24]. Cronbach’s alpha is also reported below as a measure of questionnaire reliability, but the results should be interpreted cautiously since some of the assumptions of its use (e.g. all raters are rating the same subject, object or event) are not met in this study. Its use here to check on the internal consistency of the questionnaire is complemented by a signal-to-noise ratio formula for checking the reliability of the questionnaire data [24]. The formula is designed to handle unbalanced, uncrossed and fully nested data and combines item, rater and subject variances at both the raw score and aggregated levels while also taking the average number of raters per ratee into account.

## Results

### Patient data (PQ)

The average raw patient score on all 12 questionnaire items was a high 89.16% (Table 1), indicating an overall response tending towards the higher end of the ‘very good’ to ‘excellent’ range. The highest scoring item was ‘Respect shown’ (91.61%) and the lowest ‘Reassurance’ (87.79%).

The average rate of missing responses per item throughout the entire set of patient responses was a very low 0.43% (Table 1). There was an average of 40.14 patient responses per doctor.

57.9% of patients were aged between 25 and 59 years of age, 23% were over 60, and 16.4% were under 25, with 2.8% not declaring their age. Patients under 25 gave a significantly lower average score (88.55%, *p* ≤ 0.01) than patients aged 25–59 (89.33%) and patients over 60 (89.42%), although the lowest average is still within the ‘very good’ to ‘excellent’ range. 62.8% of patients were female, 33.4% were male, and 3.8% did not declare their gender. Male patients gave a significantly lower average score (87.95%) than female patients (89.90%, *p* ≤ 0.01).

Reliability of the 12 Likert scale items was 0.73 using a one-way random ICC, indicating good agreement among the different raters on how the questionnaire items were to be interpreted. Data reliability calculated using a signal-to-noise ratio formula [25] was 0.895, in contrast to Cronbach’s alpha of 0.970 which assumes all raters are rating the same practitioner. In other words, 89.5% of the data is likely to be true data with the rest due to noise and error from interactions between raters, items and ratees.

A Kaiser-Meyer-Olkin (KMO) sampling adequacy measure of 0.98 and a significant Bartlett’s test for sphericity (*p* ≤ 0.001) of the 12 items indicated that PCA was appropriate. PCA using the varimax rotation method (to spread the highly loaded items across components) revealed two previously identified primary dimensions known to affect patient evaluations, namely, interpersonal communication and possible impediments to access to care [26], thereby establishing criterion (external) validity (Table 2). Moreover, overall satisfaction and recommendation (Q1 and Q12) were associated with interpersonal communication items, in line with previous studies (e.g. [27]) and thereby establishing construct validity of the questionnaire.

**Table 2:** PCA on patient items, with only the highest item loadings shown

Patient items	Components	
	1 (Interpersonal communication)	2 (Access to care)
Q1 Satisfaction with visit	.771	
Q2 Warmth of greeting	.603	
Q3 Ability to listen	.660	
Q4 Explanations	.766	
Q5 Reassurance	.797	
Q6 Confidence in ability	.803	
Q7 Express concerns		.684
Q8 Respect shown		.763
Q9 Time for visit		.834
Q10 Consideration		.718
Q11 Concern for patient		.730
Q12 Recommendation	.693	
*Variance explained*	*41.52%*	*37.87%*

When average patient scores were aggregated and analysed by doctor type, RACGP Registrars and AHPRA doctors received significantly lower scores (89.05 and 88.14%, respectively) than GPs-CPD and ACRRM Registrars doctors (90.90 and 90.47%, respectively, *p* ≤ 0.05).

### Colleague data (CQ)

The average raw colleague score on all 18 items was 88.35% (Table 3), indicating an overall response in the ‘very good’ to ‘excellent’ range. The highest scoring item was ‘Trustworthiness’ (93.54%) and the lowest ‘Ability to say no’ (81.02%). Colleagues who were doctors gave fewer missing responses (3.56%) and significantly lower scores (85.49%) than non-doctor colleagues (7.88, 89.87%, *p* ≤ 0.001, Table 3). Female colleagues gave more missing values and significantly higher scores (7.31 and 89.44%, respectively) than male colleagues (4.52, 86.17%, Table 3).

**Table 3:** Score given by colleagues for all 18 questionnaire items, broken down by colleague type and gender of colleague

Colleague items	All colleagues (*n* = 23,268)		Doctor colleagues (*n* = 7725)		Other colleagues (*n* = 15,245)		Female colleagues (*n* = 15,746)		Male colleagues (*n* = 7263)	
	Mean	Missing %	Mean	Missing %	Mean	Missing %	Mean	Missing %	Mean	Missing %
1Q1 Clinical Knowledge	88.36	8.9	84.70	.7	90.50	12.9	89.79	11.6	85.66	3.0
Q2 Clinical ability	88.31	11.3	85.03	2.5	90.26	15.7	89.69	14.3	85.77	4.9
Q3 Communication with patients	88.42	4.9	85.28	3.1	90.06	5.8	89.71	5.7	85.77	3.2
Q4 Compassion/empathy	89.36	4.4	86.69	2.3	90.76	5.5	90.35	5.3	87.36	2.6
Q5 Communication with colleagues	88.71	1.5	86.54	.5	89.82	2.0	89.61	1.8	86.91	.8
Q6 Punctuality and reliability	88.72	4.2	87.02	3.3	89.60	4.7	89.28	4.0	87.62	4.9
Q7 Respect for colleagues	91.43	.8	89.70	.4	92.31	1.0	92.06	.9	90.21	.6
Q8 Ability to say “no”	81.02	11.5	77.70	9.5	82.74	12.5	82.08	12.0	78.82	10.1
Q9 Awareness of limitations	87.15	7.5	84.14	2.7	88.79	9.9	88.29	9.1	84.91	4.0
Q10 Team orientation	86.61	6.3	84.19	3.6	87.89	7.6	87.48	7.0	84.84	4.7
Q11 Use of resources	87.40	11.6	84.55	5.9	88.98	14.4	88.68	13.5	84.90	7.3
Q12 Ability to manage stress	83.99	8.1	80.69	5.9	85.73	9.2	85.21	8.5	81.51	7.2
Q13 Respect for confidentiality	92.68	2.3	89.51	1.8	94.30	2.5	93.74	2.3	90.53	2.3
Q14 Appearance and behaviour	92.66	.4	89.75	.3	94.14	.4	93.78	.3	90.35	.5
Q15 Respect to their own health	88.55	10.8	84.18	9.2	90.79	11.5	90.15	10.9	85.17	10.4
Q16 Trustworthiness/honesty/probity	93.54	2.3	91.44	1.3	94.63	2.8	94.22	2.5	92.22	1.6
Q17 Management/leadership skills	83.48	16.1	80.60	10.6	85.08	18.9	84.87	17.9	80.77	12.3
Q18 Overall ability as a doctor	*89.90*	*3.0*	87.17	.6	91.33	4.3	90.95	3.9	87.82	1.2
*Averages*	*88.35*	*6.43*	*85.49*	*3.56*	*89.87*	*7.88*	*89.44*	*7.31*	*86.17*	*4.52*

Reliability of the 18 Likert scale items was 0.503 using a one-way random ICC, indicating moderate agreement among the different raters on how the questionnaire items were to be interpreted. Data reliability calculated using a signal-to-noise ratio formula [25] was 0.81, in contrast to Cronbach’s alpha of 0.95 assuming all colleagues are rating the same doctor. In other words, 81% of the data is likely to be true data with the rest due to noise and error from interactions between raters, items and ratees.

The KMO measure of sampling adequacy was a high 0.967 and Bartlett’s test of sphericity was significant (*p* ≤ 0.001). Three components explaining 67% of the variance in the data were found (Table 4, left part), corresponding to behaviour (component 1), performance (component 2) and self-management (component 3). Communication with patients is identified with the performance component and colleagues’ overall rating of doctor ability is most strongly associated with the performance component. These three components are closely related to four previously identified categories of doctor performance: inappropriate behaviour, inappropriate use of resources, deficient competence and physician impairment [28]. The clear extraction of these three components here is in line with previously peer- established performance categories, and indicates good external and construct validity of the colleague questionnaire.

**Table 4:** Principal component analysis reveals three components underlying colleagues’ ratings, with only the highest component loadings shown for the 18 items

Colleague items	Components		
	1 (Behaviour)	2 (Performance)	3 (Self-management)
Q1 Clinical Knowledge		.784	
Q2 Clinical ability		.796	
Q3 Communication with patients		.661	
Q4 Compassion/empathy	.654		
Q5 Communication with colleagues	.619		
Q6 Punctuality and reliability	.561		
Q7 Respect for colleagues	.772		
Q8 Ability to say “no”			.795
Q9 Awareness of limitations			.524
Q10 Team orientation	.579		
Q11 Use of resources		.491	
Q12 Ability to manage stress			.600
Q13 Respect for confidentiality with patients and colleagues	.653		
Q14 Appearance and behaviour	.665		
Q15 Respect to their own health			.673
Q16 Trustworthiness/honesty/probity	.698		
Q17 Management/leadership skills		.569	
Q18 Overall ability as a doctor		.687	
*Variance explained*	*26.63%*	*22.87%*	*17.83%*

When colleague scores were aggregated and analysed by doctor type, significantly lower scores (*p* ≤ 0.001) were received by AHPRA doctors in comparison to all other doctors (86.09% versus an average of 88.47%).

### Self-evaluation data in comparison to patient and colleague scores.

#### Patient questionnaire (SEPQ)

**Fig. 2:**
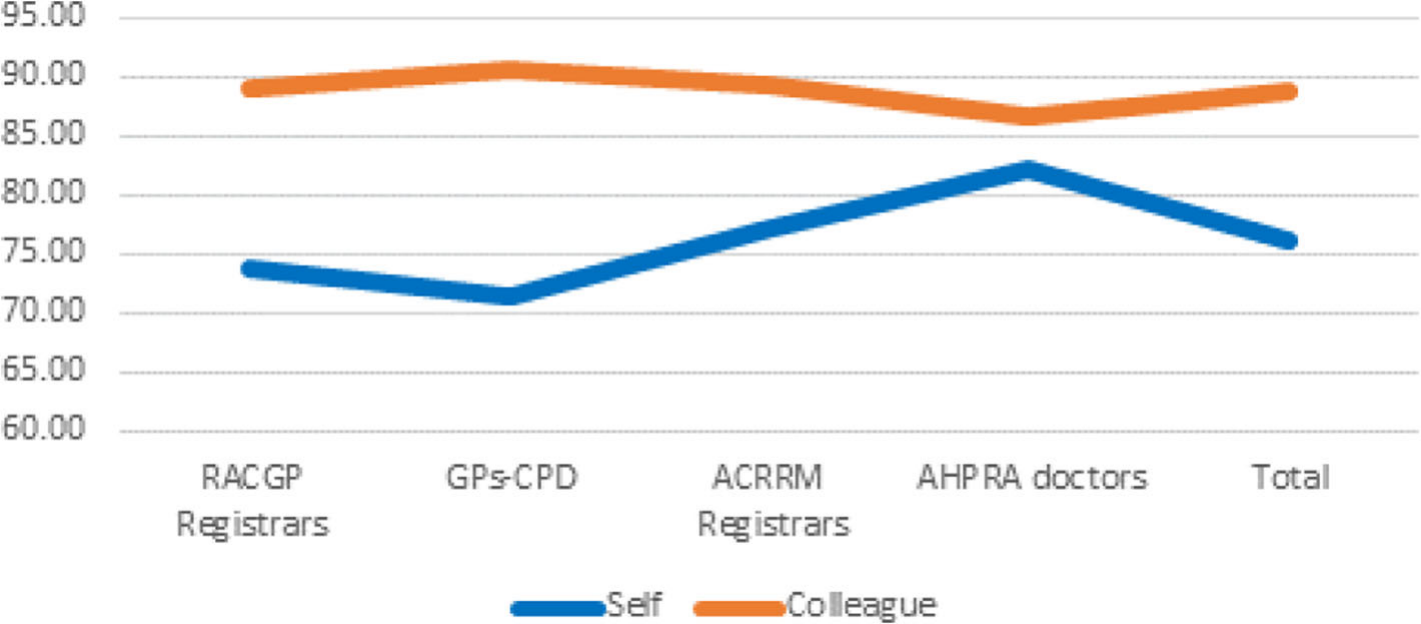
Comparison between 375 doctor self-evaluations using patient questionnaire and actual patient scores across the 12 items and overall averages, with asterisked items denoting weak (r ≤ 0.20) but significant correlations (p ≤ 0.05). Note that the y axis has been limited to the range 70–95% to make the differences clearer by item

375 doctors completed the patient questionnaire as part of self-evaluation, giving themselves an average of 78.28% in comparison to an actual patient average of 88.45% for those same doctors. Reliability across the 13 items using the one-way random ICC was 0.67, indicating good agreement among the doctors on how the questionnaire items were to be interpreted from a patient perspective. There was a weak but significant correlation in self-score and patient-score averages (*r* = 0.126, *p* = 0.015). This weak but significant overall correlation was also reflected in correlations on items Q1, Q2, Q6, Q8, Q9, Q11 and Q12 between self-score and patient score (*r* ≤ 0.20, *p* ≤ 0.05, Fig. 2).

There were significant differences in the average self-scores of RACGP Registrars (72.76%) and AHPRA doctors (80.55%, *p* ≤ 0.001), with the latter giving themselves significantly higher scores on all items (p ≤ 0.001) except item 9 (‘Amount of time’).

#### Colleague questionnaire (SECQ)

**Fig. 3:**
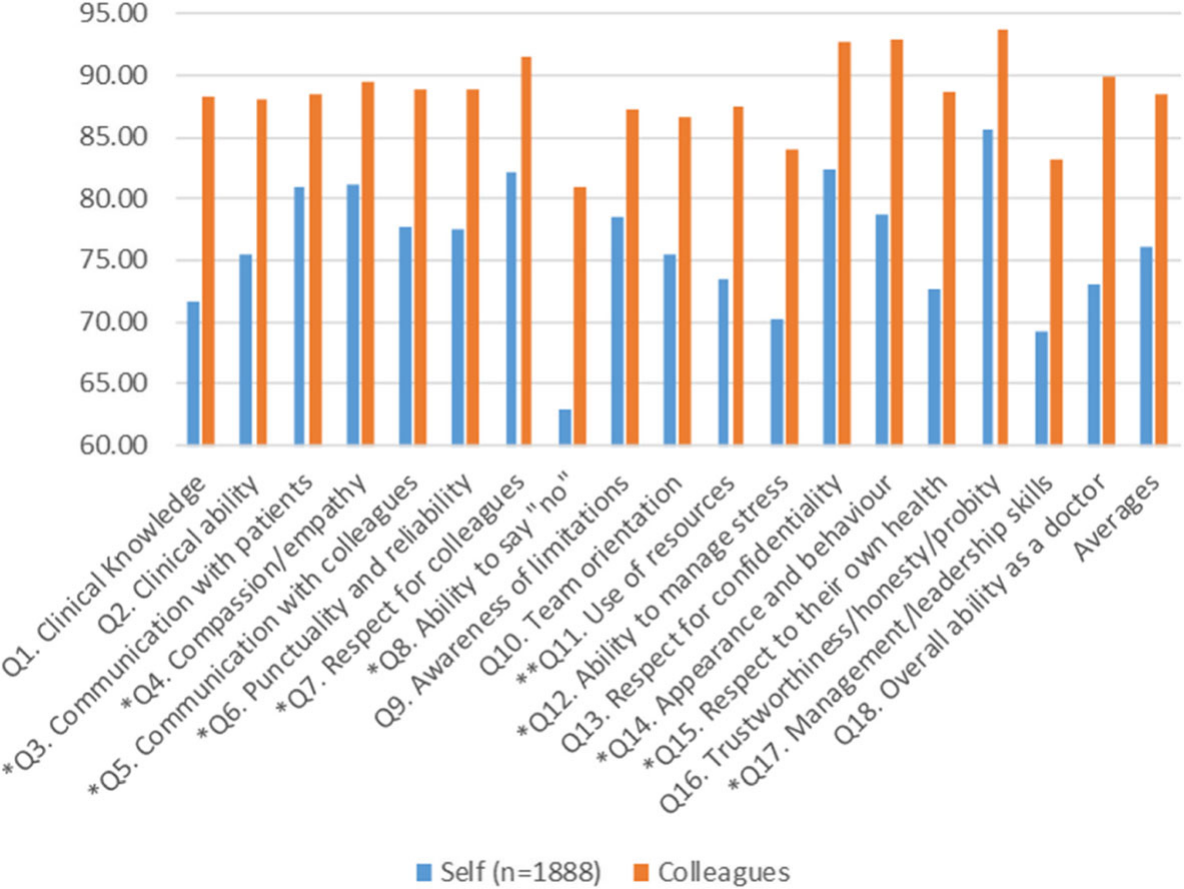
Comparison between 1888 doctor self-evaluations using the colleague questionnaire and actual colleague scores across the 18 items and overall averages, with single asterisked items denoting weak but significant positive correlations and double-asterisked weak but significant negative correlations, (all *r* ≤ 0.20, *p* ≤ 0.05). Note that the y axis has been limited to the 60–95% range to make the differences clearer by item

1888 doctors completed the colleague questionnaire as part of self-evaluation, giving themselves an average of 76.05% in comparison to an actual colleague average of 88.37% for those same doctors. Single measures ICC was 0.47, indicating moderate agreement about how to interpret the items. There was no correlation in self-score and colleague score averages. However, there were weak positive but significant correlations on individual items Q3-Q8, Q12, Q14, Q15, and Q17, and one weak negative but significant correlation on Q11 (all *r* ≤ 0.20, *p* ≤ 0.05, Fig. 3).

A small number of doctors (39) declared their own gender when completing the colleague questionnaire (16 female, 23 male), with female doctors tending to given themselves an average 5% lower score than male doctors (81.15%). However, this difference between self-declared doctors was not significant (*p* = 0.167).

**Fig. 4:**
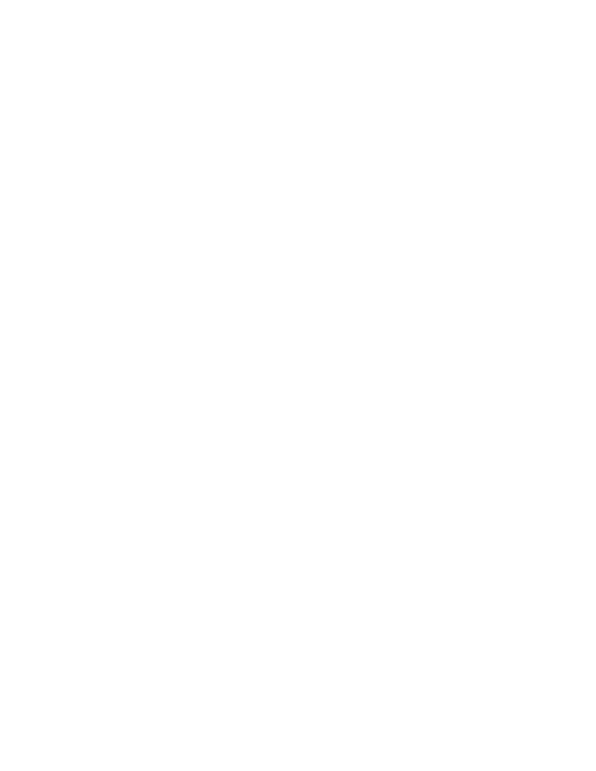
AHPRA doctors give themselves significantly higher scores and receive significantly lower scores from colleagues using the colleague questionnaire (RACGP Registrars *n* = 1042, GPs-CPD *n* = 14, ACRRM Registrars *n* = 547, AHPRA doctors *n* = 285, Total *n* = 1888). Note that the y axis has been limited to the range 60–95% to make the differences clearer by doctor group

When overall averages were broken down by type of doctor, AHPRA doctors gave a significantly higher score to themselves than all other doctor types and also received significantly lower scores than all other doctor types from colleagues (p ≤ 0.05, Fig. 4).

### MSF analysis.

#### Patient, colleague and colleague self-evaluation data (PQ, CQ and SECQ)

1228 doctors had patient scores, colleague scores and self-evaluation using the colleague questionnaire. For these 1228 doctors there was a moderate and significant *convergence* (positive correlation, or the degree to which two measures move together) between the overall average scores awarded by colleagues (88.29%) and patients (89.58, *r* = 0.305, *p* ≤ 0.001).

PCA of all 48 items (12 patient items, 18 colleague items, 18 colleague self-evaluation items) revealed three main components explaining 68.42% of the variance in the data. These three components separate the three sets of questionnaire items cleanly (patient items explaining 23.25% of the variance, colleague items 24.79% and colleague self-evaluation items 20.38%), indicating structural and construct validity. That is, the three questionnaires are non-redundantly measuring three different aspects of doctors. This was confirmed by a high KMO measure of 0.97 and significant Bartlett’s test of sphericity (*p* ≤ 0.001).

#### Patient, colleague, colleague self-evaluation and patient self-evaluation.

Three hundred sixty seven doctors had patient scores, colleague scores, self-evaluation using the colleague questionnaire and self-evaluation using the patient questionnaire (60 items) For these 367 doctors, there was a high degree of convergence between their self-evaluation colleague and self-evaluation patient scores (*r* = 0.875, *p* ≤ 0.001), and weak but significant convergence between self-evaluation patient scores and actual scores received from patients (*r* = 0.126, *p* ≤ 0.05).

PCA of all 60 items revealed three main components explaining 68.13% of variance. Two of the components clearly separated colleague-scored items (20.93% of variance) and patient-scored items (18.63%), with the third component containing all the self-evaluation items (colleague and patient, 28.57%). That is, the 30 self-evaluation items are measuring the same component in contrast to the 30 items scored by colleagues and patients. This was confirmed by a high KMO measure of 0.958 and sphericity test (*p* ≤ 0.001).

## Discussion

Doctors received high satisfaction scores from patients (average 89%) and colleagues (88%), with GPs-CPD and ACCRM Registrars (Groups 2 and 3) receiving significantly higher average scores from patients (91 and 90%, respectively), and AHPRA doctors (Group 4) significantly lower scores from colleagues (86%). Since Group 4 doctors had been previously recognized as doctors possibly requiring further support, the results here provide additional evidence of external (population) validity and indicate that MSF can help to identify specific aspects of relative under-performance for enhanced professional development.

Overall, voluntary (Group 2) versus non-voluntary (Groups 1, 3 and 4) participation had no effect on scores in this study. Patients rated doctors highest on respect shown (92%) and lowest on reassurance provided (88%). Colleagues rated doctors highest on trustworthiness (94%) and lowest on ability to say ‘no’ (81%).

PCA revealed two dimensions to the patient questionnaire (interpersonal communication, impediments to access to care) which have also been identified in previous studies, thereby confirming their importance in patient evaluation. Similarly, PCA revealed three dimensions previously identified with doctor performance: behaviour, performance and self-management. These dimensions demonstrate good and robust external and construct validity of the patient and colleague questionnaires used in this study. Data reliability as measured by signal-to-noise ratios was 0.895 for the patient data and 0.81 for the colleague data, indicating limited interaction errors for the convenience sampling method adopted for collecting the data. ICCs and Cronbach’s alpha showed agreement in item interpretation and internal consistency of questionnaires.

With regard to self-evaluation, doctors gave themselves a 10% lower score on the patient questionnaire (average 78%) than they received from patients (88%), with weak but positive correlations on seven of the 12 items. There is therefore some evidence from this study of convergence of agreement between patient scores received and doctor self-evaluation from a patient perspective on many aspects of interpersonal communication and access to care. Similarly, doctors gave themselves a 12% lower score (average 76%) than actual scores received from colleagues (88%). Again, there is some evidence of convergence of agreement between colleague scores received and doctor self-evaluation from a colleague perspective across all three dimensions of behaviour, performance and self-management. The differences of 10 and 12% are consistent with findings that doctors may feel more comfortable with reflection related to patient medical issues than team (colleague) domain issues [18]. This was partly confirmed by the difference in single measures ICCs, where doctors tended to agree more on how to interpret the patient items (0.67) in contrast to the colleague items (0.47). Given that self-evaluations may generally tend to result in scores lower than scores actually received, helping doctors feel more at ease with self-evaluation from a colleague perspective could form part of future mentoring support to narrow the gap between patient-based and colleague-based scores as well as help doctors interpret colleague items more consistently. Such mentoring may enhance the ability of doctors in identifying suitable strategies for self-improvement and self-management by making them more comfortable with the outcomes of peer-review from a colleague perspective.

MSF analysis using the three evaluation measures of patient scores, colleague scores and self-evaluation using the colleague questionnaire (270° MSF) showed significant convergence (positive correlation), with PCA clearly revealing the three separate instrument dimensions. Adding the fourth instrument dimension of self-evaluation using the patient questionnaire (360° MSF) showed two types of convergence: (i) between self-evaluation using the patient questionnaire and self-evaluation using the colleague questionnaire, and (ii) between self-evaluation patient scores and actual patient scores. PCA revealed that, for 360° MSF, all the self-evaluation items (patient and colleague) formed one component whereas the other two components clearly separated colleague-scored and patient-scored items. In other words, this study has shown that one round of self-evaluation using either the patient or colleague questionnaire is sufficient for MSF purposes, with the choice of which instrument to use depending on the importance placed on doctors evaluating themselves through their patients’ or colleagues’ perspectives. However, given that doctors may find it more difficult to evaluate themselves from a colleague perspective than from a patient perspective, it may be wise to consider introducing mechanisms that help doctors feel as comfortable with evaluating themselves from a colleague perspective as from a patient’s to help reduce the small differences in self-evaluation scores that this study has revealed. Reducing this difference may also help to enhance the convergence between colleague scores and self-scores using the colleague questionnaire.

## Conclusions

The questionnaires used in this study are fit for purpose and can be used for MSF involving patient feedback and colleague feedback (180° MSF), supplemented with self-evaluations using the patient and colleague questionnaires (a further 180° MSF), as appropriate. Self-evaluations can expect to return responses that are at least 10% below actual scores received, with doctors agreeing on how to interpret patient items more than colleague items. However, neither self-evaluation could be separated from each other as separate MSF dimensions, leading to the conclusion that 270° MSF may be sufficient for CPD under the MBA’s new framework. If self-evaluations are administered, it is recommended that the colleague questionnaire be used since colleague items cover patient care aspects and include aspects of clinical competence, clinical knowledge, self-management and professional behaviour. Mentoring programmes should also be designed to help doctors feel more comfortable about evaluating themselves from a colleague perspective so that discrepancies between self and colleague evaluation can lead to improved strategies for performance improvement. Finally, it should be noted that all doctor groups received aggregated scores in the ‘very good’ to ‘excellent’ range. The relative findings reported here must be interpreted in that context.

Limitations of this study include the variable numbers of doctors used for each part of the analysis due to data being collected at different times for such a large-scale study. Also, the large sample sizes involved can lead to small differences being statistically significant. With smaller sample sizes, a much bigger difference between item averages would have been necessary to identify truly significant differences. Finally, doctors selected which colleagues will provide feedback, which may result in higher than normal colleague evaluation scores.

## Abbreviations

ACRR: Australian College of Rural & Remote Medicine
AHPRA: Australian Health Practitioner Regulation Agency
ANOVA: Analysis of variance
CFEP: Client Focused Evaluation Program pty
CPD: Continuing professional development
CQ: Colleague questionnaire
GMC: General Medical Council (UK)
GP: General practitioner
GPs-CPD: General practitioners undertaking MSF as CPD activity
ICC: Intraclass correlation coefficient
KMO: Kaier-Meyer-Olkin
MBA: Medical Board of Australia
MSF: Multisource feedback
PCA: Principal component analysis
PPF: Professional Performance Framework
PQ: Patient questionnaire
RACGP: Royal Australian College of General Practitioners
RTO: Regional Training Organisation
SECQ: Doctor self-evaluation using CQ
SEPQ: Doctor self-evaluation using PQ
SPSS: Statistical Package for the Social Sciences

## References

[1] Chaudhry H, Rhyne J, Waters S, Cain FE, Talmage L (2012). Maintenance of licensure: evolving from framework to implementation. J Med Regul.

[2] Iglehart JK, Baron RB (2012). Ensuring physicians’ competence – is maintenance of certification the answer?. N Engl J Med.

[3] Swankin D, LeBuhn RA, Morrison R. Implementing continuing competency requirements for health care practitioners. AARP #2006–16, July 2006. https://assets.aarp.org/rgcenter/health/2006_16_competency.pdf (accessed on 8 January 2018).

[4] Nasca TJ, Philibert I, Brigham T, Flynn TC (2012). The next GME accreditation system--rationale and benefits. N Engl J Med.

[5] Zhao L, Sun T, Sun B-J, Zhao Y-H, Norcini J, Chen L (2015). Identifying competencies of doctors in China. BMC Med Ed.

[6] Professional Competence: Guidelines for doctors. The Medical Council of the Republic of Ireland 2011. https://www.medicalcouncil.ie/Existing-Registrants-/Professional-Competence/Professional-Competence-Guidelines.pdf. Accessed 8 Jan 2018.

[7] Good Medical Practice The UK General Medical Council. 2013. https://www.gmc-uk.org/static/documents/content/Good_medical_practice_-_English_1215.pdf. Accessed 8 Jan 2018.

[8] Expert Advisory Group on Revalidation: Final Report. Medical Board of Australia. 2017. http://www.medicalboard.gov.au/documents/default.aspx?record=WD17%2f24295&dbid=AP&chksum=Txmn8C7v%2bC53Wjsz3sXn2w%3d%3d. Accessed 10 Jan 2018 from http://www.medicalboard.gov.au/Registration/Professional-Performance-Framework.aspx.

[9] Building a Professional Performance Framework Medical Board of Australia. 2017. http://www.medicalboard.gov.au/documents/default.aspx?record=WD17%2f24293&dbid=AP&chksum=GO%2b6DZkJeoSSVVg%2fxcDoMQ%3d%3d. Accessed 10 Jan 2018 from http://www.medicalboard.gov.au/Registration/Professional-Performance-Framework.aspx.

[10] Supporting information for appraisal and revalidation. The UK General Medical Council. 2012. https://www.gmc-uk.org/static/documents/content/RT___Supporting_information_for_appraisal_and_revalidation___DC5485.pdf_55024594.pdf. Accessed 8 Jan 2018.

[11] Ramsey PG, Wenrich MJ, Carline JD, Inui TS, Larson EB, Logerfo JP (1993). Use of peer ratings to evaluate physician performance. J Am Med Assoc.

[12] Greco M, Brownlea A, McGovern J (2001). Impact of patient feedback on the interpersonal skills of GP registrars: results of a longitudinal study. Med Educ.

[13] Violato C, Marini A, Toews J, Lockyer J, Fidler H (1997). Using peers, consulting physicians, patients, coworkers and self to assess physicians. Acad Med.

[14] Wood L, Hassell A, Whitehouse A, Bullock A, Wall D (2006). A literature review of MSF systems within and without health services, leading to ten tips for their successful design. Med Teach..

[15] Davies H, Archer J, Bateman A, Dewar S, Crossle J, Grant J, Southgate L, Specialty-specific MSF (2008). Assuring validity, informing training. Med Educ.

[16] Evans AW, McKenna C, Oliver M (2002). Self-assessment in medical practice. J Roy Soc Med.

[17] Mann K, Gordon J, MacLeod A (2009). Reflection and reflective practice in health professions education: a systematic review. Adv Health Sci Educ Theory Pract.

[18] Bindels E, Verberg C, Scherpbier A, Heeneman S, Lombarts K (2018). Reflection revisited: how physicians conceptualize and experience reflection in professional practice – a qualitative study. BMC Med Educ.

[19] Hall W, Violato C, Lewkonia R, Lockyer J, Fidler H, Toews J, Jenett P, Donoff M, Moores D (1999). Assessment of physician performance in Alberta: the physician achievement review. CMAJ.

[20] Campbell JL, Richards SH, Dickens A, Greco M, Narayanan A, Brearly S (2008). Assessing the professional performance of UK doctors: an evaluation of the utility of the general medical council patient and colleague questionnaires. Qual Safety Health Care.

[21] Overeem K, Lombarts MJ, Arah OA, Klazinga NS, Grol RP, Wollersheim HC (2010). Three methods of multi-source feedback compared: a plea for narrative comments and coworkers’ perspectives. Med Teach.

[22] Campbell JM, Roberts M, Wright C, Hill J, Greco M, Taylor M, Richards S (2011). Factors associated with variability in the assessment of UK doctors’ professionalism: analysis of survey results. BMJ.

[23] Overeem K, Wollersheim HC, Arah OA, Cruijsberg JK, Grol RPTM, Lombarts KMUMH (2012). Evaluation of physicians’ professional performance: an iterative development and validation study of multisource feedback instruments. BMC Health Serv Res.

[24] Landis JR, Koch G (1977). The measurement of observer agreement for categorical data. Biometrics.

[25] Narayanan A, Greco M, Powell H, Coleman L (2013). The reliability of big ‘patient satisfaction’ data. Big Data.

[26] Tucker J, Adams SR (2001). Incorporating patients’ assessment of satisfaction and quality: an integrative model of patients’ evaluations of their care. Man Service Qual.

[27] Kaigas, T. Assessment of the performance of practicing physicians in Canada. 5th International Medical Workforce Conference, Sydney, 2000 (Session 2); pp. 101–19. Available from http://crmcc.medical.org/publicpolicy/conference5.php. Accessed 26 Jan 2018.

[28] Overeem K, Wollersheimh HC, Arah OA, Cruijsberg JK, Gral RPTM, Lombarts KMJMH (2012). Factors predicting doctors’ reporting of performance change in response to multisource feedback. BMC Med Educ..

