# Correction to: Multisource feedback as part of the medical board of Australia’s professional performance framework: outcomes from a preliminary study

**DOI:** 10.1186/s12909-019-1490-5

**Published:** 2019-03-04

**Authors:** Ajit Narayanan, Elizabeth A. Farmer, Michael J. Greco

**Affiliations:** 1 Computer and Mathematical Sciences, School of Engineering, Auckland University of Technology, 2-14 Wakefield Street, Auckland, 1010 New Zealand; 2 Graduate Medicine, University of Wollongong NSW, Keiraville, Australia; 3 School of Medicine, Gold Coast Campus, Griffith University, Southport, Australia

**Correction to: BMC Med Educ**

**DOI 10.1186/s12909-018-1432-7**

Following publication of the original article [1], the author reported that Fig. 4 was missing. This has now been corrected in the original article.

**Fig. 4:**
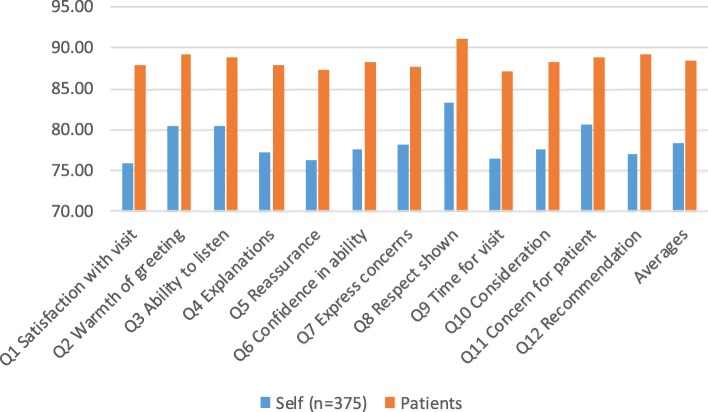
Comparison between 375 doctor self-evaluations using patient questionnaire and actual patient scores across the 12 items and overall averages, with asterisked items denoting weak (r ≤ 0.20) but significant correlations (*p* ≤ 0.05). Note that the y axis has been limited to the range 70–95% to make the differences clearer by item
